# A new species of *Dichelyne* (Nematoda: Cucullanidae) parasitizing fish of the family Pimelodidae in the Amazon estuary, northern Brazilian coast

**DOI:** 10.1590/S1984-29612026020

**Published:** 2026-07-06

**Authors:** Gabrielly Pantoja Alves, Ricardo Luis Sousa Santana, Luis Augusto Araújo dos Santos Ruffeil, Elane Guerreiro Giese, Raul Henrique da Silva Pinheiro

**Affiliations:** 1 Universidade Federal Rural da Amazônia - UFRA, Instituto da Saúde e Produção Animal – ISPA, Laboratório de Histologia e Embriologia Animal – LHEA, Belém, PA, Brasil; 2 Universidade Federal Rural da Amazônia – UFRA, Instituto da Saúde e Produção Animal, Programa de Pós-graduação em Saúde e Produção Animal na Amazônia, Belém, PA, Brasil; 3 Escola Estadual de Ensino Técnico do Pará Dr. Celso Malcher, Belém, PA, Brasil

**Keywords:** Morphology, Nematoda, Cucullanidae, parasite, fish, Amazon, Morfologia, Nematoda, Cucullanidae, parasito, peixe, Amazônia

## Abstract

A new cucullanid, *Dichelyne* (*Dichelyne*) *jutubensis* n. sp., collected from the intestine of *Propimelodus eigenmanni* (Van der Stigchel, 1946), from the Amazon estuary on the northern coast of Brazil, is described based on light and scanning electron microscopy. The prevalence of infection was 39.6% (36 infected fish/ 91 examined). Morphologically, the nematodes lacked a ventral sucker, and the number of caudal papillae in males identifies them as members of the nominotypical subgenus *Dichelyne* of the genus *Dichelyne*. The new species differs from its congeners by the presence of a conical, spiny appendage at the tip of the tail and in the number and distribution of its 10 pairs of caudal papillae: six pre-cloacal, one adcloacal, and three post-cloacal. This new species represents the fourth record of the genus in Brazilian territory and the first record for estuarine waters in northern Brazil, with *Propimelodus eigenmanni* registered as a new host for the genus.

## Resumo

Um novo cucullanídeo, *Dichelyne* (*Dichelyne*) *jutubensis* n. sp., coletado do intestino de *Propimelodus eigenmanni* (Van der Stigchel, 1946), do estuário amazônico, da costa norte do Brasil, é descrito com base em microscopia de luz e eletrônica de varredura. A prevalência de infecção foi de 39,6% (36 peixes infectados/ 91 examinados). Morfologicamente, os nematódeos apresentaram ausência de ventosa ventral e o número de papilas caudais nos machos os alocam ao subgênero nominotípico *Dichelyne*, do gênero *Dichelyne*. A nova espécie difere de seus congêneres por apresentar na ponta da cauda um apêndice cônico com espinhos e pelo número e distribuição das 10 papilas caudais: seis pares pré-cloacais, um par adcloacal e três pares pós-cloacais. Esta nova espécie representa o quarto registro do gênero para o território brasileiro e o primeiro registro para águas estuarinas do norte do Brasil, sendo *Propimelodus eigenmanni* registrado como um novo hospedeiro para o gênero.

## Introduction

Nematodes of the family Cucullanidae Cobbold, 1864 are diverse and commonly occur in the digestive tract of marine, estuarine, and freshwater fish (Timi et al., 1997; Caspeta-Mandujano et al., 2000; Moravec et al., 1997, 2001; González-Solís et al., 2002; Moravec & Justine, 2011). *Cucullanus* Müller, 1777 and *Dichelyne* Jägerskiöld, 1902 are the most representative genera, considering the number of valid species, and are pose taxonomic challenges (Pereira & Luque, 2016; Pinheiro et al., 2024). Although the genera contain numerous species, the high number of inadequate descriptions and the rather uniform morphology of cucullanids make detailed comparisons among all of them impossible and represent a real challenge for taxonomists who deal with these parasites (Moravec et al., 1993; Vieira et al., 2015).

*Dichelyne* comprises the 3 subgenera *Dichelyne* Jägerskiöld, 1902, *Cucullanellus* Törnquist, 1930, and *Neocucullanellus* Yamaguti, 1941 (Anderson et al., 2009). The subgenus *Dichelyne* comprises approximately 30 species (Worms, 2025), of which only three have been recorded for Brazil, parasitizing freshwater and marine fish: *Dichelyne* (*Dichelyne*) *leporini* Petter, 1989, parasites of Anostomidae fish (*Leporinus friderici* Bloch, 1794; *Leporinus lacustris* Amaral Campos, 1945, and *Schizodon fasciatus* Spix & Agassiz, 1829), *Dichelyne* (*Dichelyne*) *pimelodi* Moravec, Kohn & Fernandes, 1997, a parasite of Pimelodidae fish (*Pimelodus maculatus* Lacepède, 1803, and *Pimelodus* sp.), both described in the state of Paraná, and *Dichelyne* (*Dichelyne*) *bonacii* Gonzalis-Solis, Arqaez-Garcia & Guilen-Hernandez, 2002, parasite of Lutjanidae fish (*Lutjanus griseus* Linnaeus, 1758, in *Lutjanus analis* Cuvier, 1828 and *Rhomboplites aurorubens* Cuvier, 1829, in the state of Sergipe (Luque et al., 2011).

When compiling their checklist of nematodes parasitizing fish in the Brazilian Amazon (Santos Reis et al., 2021), did not record the presence of the genus *Dichelyne* in the region. During a parasitological examination of Pimelodidae fishes from the Amazon River estuary (state of Pará), several nematodes were recovered. A detailed morphological study of these specimens, based on light and scanning electron microscopy, revealed that they represent a new species of *Dichelyne* (*Dichelyne*).

## Material and Methods

Ninety-one specimens of *Propimelodus eigenmanni* (Siluriformes: Pimelodidae) were captured by artisanal fishers from the island of Jutuba (Marajó Bay 0°47'54.7"S 48°24'10.7"W), State of Pará, Brazil. Fish were collected from March to May 2025 and transported dead on ice to the Laboratório de Histologia e Embriologia Animal, Instituto da Saúde e Produção, Universidade Federal Rural da Amazônia, City of Belém, state of Pará. All applicable institutional, national, and international guidelines for animal care and use were followed (permission number CEUA: 9243170321; SISBIO: 68028-4). In the laboratory, the digestive tract of each specimen was separated and placed in Petri dishes with saline and examined using a stereomicroscope. Live nematodes were recovered from the intestine of *Propimelodus eigenmanni* specimens, washed in saline solution and then fixed in a hot ethanol–formaldehyde–acetic acid solution (930 mL of 70% ethanol, 50 mL of commercial formaldehyde, and 20 mL of acetic acid) and preserved in 70% ethanol. They were then examined using light microscopy and scanning electron microscopy following procedures described by Pinheiro et al. (2018). All measurements are presented in millimeters, unless otherwise indicated. The system adopted for describing male caudal papillae is according to Moravec et al. (1993), Timi et al. (1997), Moravec et al. (1997) and González-Solís et al. (2002). The fish nomenclature adopted was according to FishBase (Froese & Pauly, 2025). The type material was deposited in the Coleção de Invertebrados não Arthropoda of the Museu Paraense Emílio Goeldi (MPEG), municipality of Belém, State of Pará, Brazil: Holotype male (MPEG 000428), allotype female (MPEG 000429) and 8 paratypes (4 males: MPEG 000430; 4 females: MPEG 000431).

## Results

A total of 80 nematodes were recovered from the intestine of *Propimelodus eigenmanni*, with a prevalence of 39.6%, (36 infected fish/91 examined) mean intensity of infection 2.5, mean abundance of 0.88, and range of infection of from 1 to 15 nematodes adults per host. All specimens collected showed characteristics compatible with the genus *Dichelyne* but could not be attributed to a known species, meaning that a new species is described here. The morphological and morphometric characteristics of this new species are presented below and in Table 1.

**Table 1:** Morphometric characteristics of *Dichelyne (Dichelyne) jutubensis* n. sp. parasite of *Propimelodus eigenmanni* in Brazil.

**Caracteres**	***Dichelyne* (*D.*) *jutubensis* n. sp.**		***Dichelyne (D.) leporini***		***Dichelyne (D.) pimelodi***		***Dichelyne (D.) bonacii***	
	**Male**	**Female**	**Male**	**Female**	**Male**	**Female**	**Male**	**Female**
**Host**	***Propimelodus eigenmanni***		*Leporinus friderici*		*Pimelodus* sp.		*Lutjanus analis*	
**Group of hosts**	**(Siluriformes)**		(Siluriformes)		(Siluriformes)		(Perciformes)	
**Locality**	**Pará, Brazil**		Paraná, Brazil		Paraná, Brazil		Sergipe, Brazil	
**Environment**	**Brackish water**		Freshwater		Freshwater		Marine	
**Length**	**2−5**	**4−8**	9.72	17.71−23.87	6.90	Unknow	11.2	13.4
**Nerve ring**	**0.167−0.293**	**0.168−0.293**	0.272	0.340−0.367	0.340	−	0.386−0.455	0.495−0.552
**Deirids**	**0.303−0.677**	**0.373−0.733**	0.911	1.16−1.35	0.979	−	0.584−0.986	0.425−0.552
**Excretory pore**	**0.343−0.837**	**0.600−0.967**	−	1.05−1.28	1.14	−	0.693−1.096	0.861−1.198
**Esophastome**	**0.170−0.277**	**0.202−0.313**	0.218	0.272**−**0.313	0.218	−	0.297−0.396	0.336−0.467
**Esophagus**	**0.477−0.670**	**0.557−0.790**	1.14	1.41−1.65	0.911	−	0.87−1.049	0.930−1.292
**Caecum**	**0.117−0.333**	**0.267−0.463**	0.925	1.20**−**1.27	0.286		0.396−523	0.495−0.688
**Vulva (Position)**	**Postequatorial**		Postequatorial		−		Postequatorial	
**Vulva**	**1−4**		12.88−15.01		−		3.689−5.380	
**Precloacal papillae**	**6 (4sv+1L+1dL)+1u**		5		4sv+1u		6 (3sv+1sv+1SL+1L)+1u	
**Adcloacal papillae**	**1ad**		1		2 (1sv−1L)		2sv	
**Postcloacal papillae**	**3 (2sv+1dL)+1ph**		4		4 (2sv−2L)		2 (1sv+1SL)+1ph	
**Right spicule**	**0.587−1.093**		0.660		1.46		0.45	
**Left spicule**	**0.547−1.027**		**−**		**−**		0.65	
**Gubernaculum**	**0.050−0.088**		0.090		0.128		0.11	
**Tail**	**0.100−0.148**	**0.137−0.208**	0.218	0.367−0.422	0.231	−	0.19	0.19−0.30
**Reference**	**Present study**		Moravec et al. (1993)		Moravec et al. (1997)		Alves et al. (2019)	

Abbreviations: sv= subventral papillae; L= lateral papillae; dL= dorsolateral papillae; SL= sublateral papillae; ph=phasmids; ad= adcloacal papillae and u= unipared papillae.

### Description

**Figure 1:**
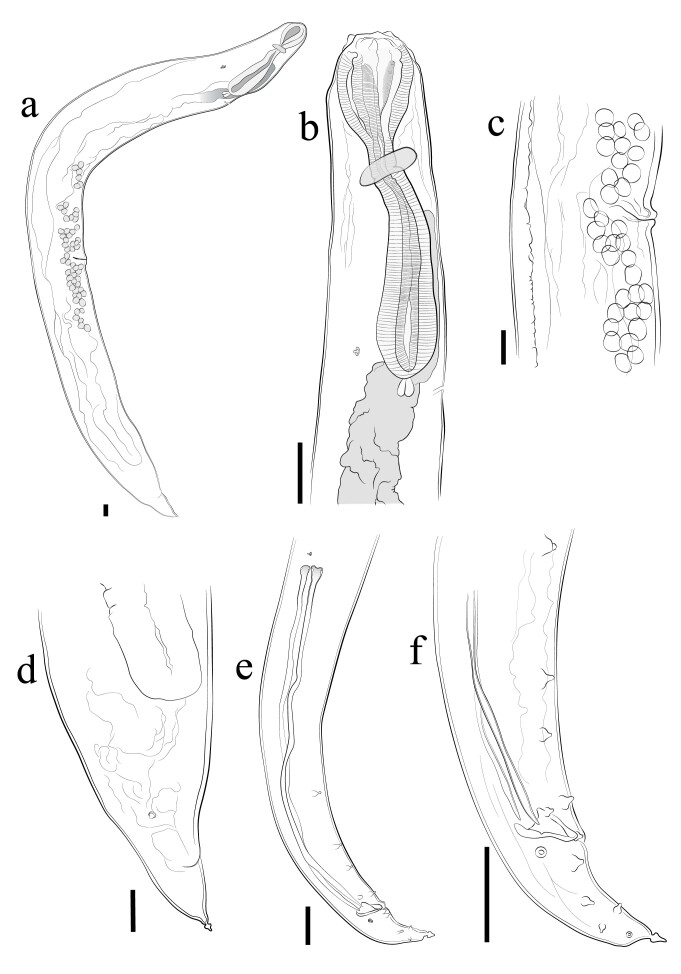
*Dichelyne* (*Dichelyne*) *jutubensis* n. sp., from *Propimelodus eigenmanni*, eastern Amazon: (a) female ventral view; (b) Anterior end of female, ventral view; (c) Region of vulva, lateral view; (d) Posterior end of female, lateral view; (e, f) Posterior end of male, lateral view, respectively. Scale bars: (a, b, c, d, e, f) = 20 μm.

**Figure 2:**
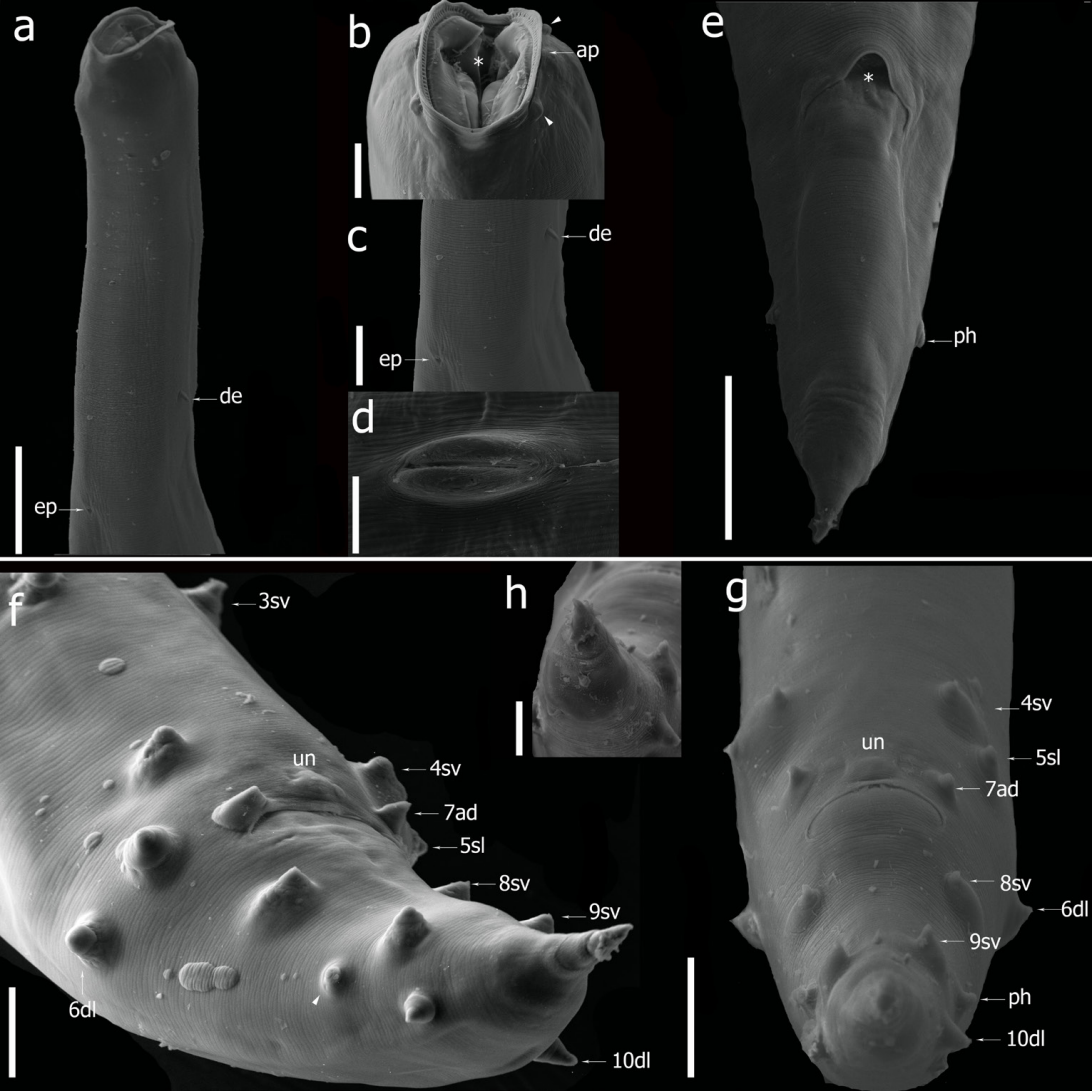
*Dichelyne* (*Dichelyne*) *jutubensis* n. sp., from *Propimelodus eigenmanni*, eastern Amazon, scanning electron micrographs: (a, b) Cephalic region, lateral and apical views, respectively: deirids (de), excretory pore (ep), papillae (arrowheads), amphids (ap), oral opening (*); (c) Deirid and excretory pore, lateral view; (d) Vulva, ventral view; (e) Tail of female, ventral view: anus (*) and phasmids (arrow); (f, g) Posterior extremity of male, lateral and ventral view; (h) Tail tip, apical view (detail of spines). Scale bars: (a) = 100 μm; (c, e) = 50 μm; (d, f, g) = 20 μm; (h) = 10 μm. Abbreviations: papillae (p1-p10), phasmid (ph), and unpaired papilla (un).

***General***. Medium-sized nematode, opaque white when alive. Cuticle finely striated transversally. Cephalic region slightly asymmetrical in lateral view; without alae (Figures 1a; 2a). Cephalic end rounded, oral aperture dorsoventrally elongate, slit-like, surrounded by raised narrow collarette, bearing row of small denticles on inner surface with row of *c.* 50 (Figure 2b). Cephalic end with four submedian papillae and pair of lateral amphids (Figures 1b; 2b). Nerve ring surrounds esophagus in the final portion of the esophastome (Figure 1b). Deirids are small and pointed (Figures 1b; 2a, c). Excretory pore below the esophagus (Figures 1b; 2a, c). The esophagus muscular, expanded at anterior end forming well-developed pseudobuccal capsule (the esophastome) (Figure 1b). Single ventral intestinal caecum (Figure 1b) of variable length present. Posterior end of the esophagus expanded; esophageal valve well-developed, nonsclerotized, opening into intestine.

**Male (based on 20 specimens, holotype measurements in parentheses):** The body length 4 mm ± 1 (2–5 mm) [3.65 mm], maximum width at the junction between esophagus and intestine 0.224 mm ± 66 (0.143-0.314 mm) [0.200 mm]. Nerve ring, deirids, and excretory pore 0.196 mm ± 16 (0.167–0.293 mm) [0.193 mm], 0.483 mm ± 92 (0.303–0.677 mm) [0.450 mm], and 0.597 mm ± 154 (0.343–0.837 mm) [0.570 mm], respectively, from anterior end of body. Esophastome 0.227 mm ± 31 (0.170–0.277 mm) [0.240 mm] long and 0.091mm ± 19 (0.063–0.123 mm) [0.096 mm] wide. Esophagus 0.551 mm ± 57 (0.447–0.670 mm) [0.536 mm] long and 0.088 mm ± 20 (0.063–0.140 mm) [0.086 mm] wide, representing 16% (11–20%) [14%] of total the body length. Intestinal caecum of variable length 0.222 mm ± 117 (0.117–0.333 mm) [0.256 mm] long, 0.059 mm ± 16 (0.033–0.090 mm) [0.056 mm] wide, representing 40% (26–54%) [47%] of esophageal length. Posterior end of body notably curved ventrally. Cloacal opening without protruding lips (Figures 2f, g). Postdeirids 1 mm ± 0 (0.700–2 mm) [1 mm]. Ten pairs of caudal papillae: six precloacal pairs (1st, 2nd, 3rd, 4th pairs subventral, 5th pair lateral, 6th pair dorsolateral), 7th pair adcloacal papillae and three postcloacal pairs (pairs 8th and 9th subventral, 10th pair dorsolateral); a pair of lateral papilla-like phasmids located between pairs 8th and 9th (Figures 1e; 2f, g). Unpaired ventral papilla located slightly anterior to cloaca (Figures 2f, g). Spicules filiform, long, subequal and weakly sclerotized, right spicule 0.883 mm ± 137 (0.587–1.093 mm) [0.793 mm] long, left spicule 0.819 mm ± 121 (0.547–1.027 mm) [0.773 mm] long, representing 25% (17–33%) [21%] of body length (Figure 1e); ventral sucker absent. Gubernaculum weakly sclerotized and Y-shaped, narrow in lateral view, 0.070 mm ± 7 (0.050–0.088 mm) [0.068 mm] long (Figure 1f). Tail 0.124 mm ± 15 (0.100–0.148 mm) [0.123 mm] long, with the presence of a crown adorned with approximately 15 small spines (Figure 2h). Caudal alae absent (Figures 2f, g).

**Figure 3:**
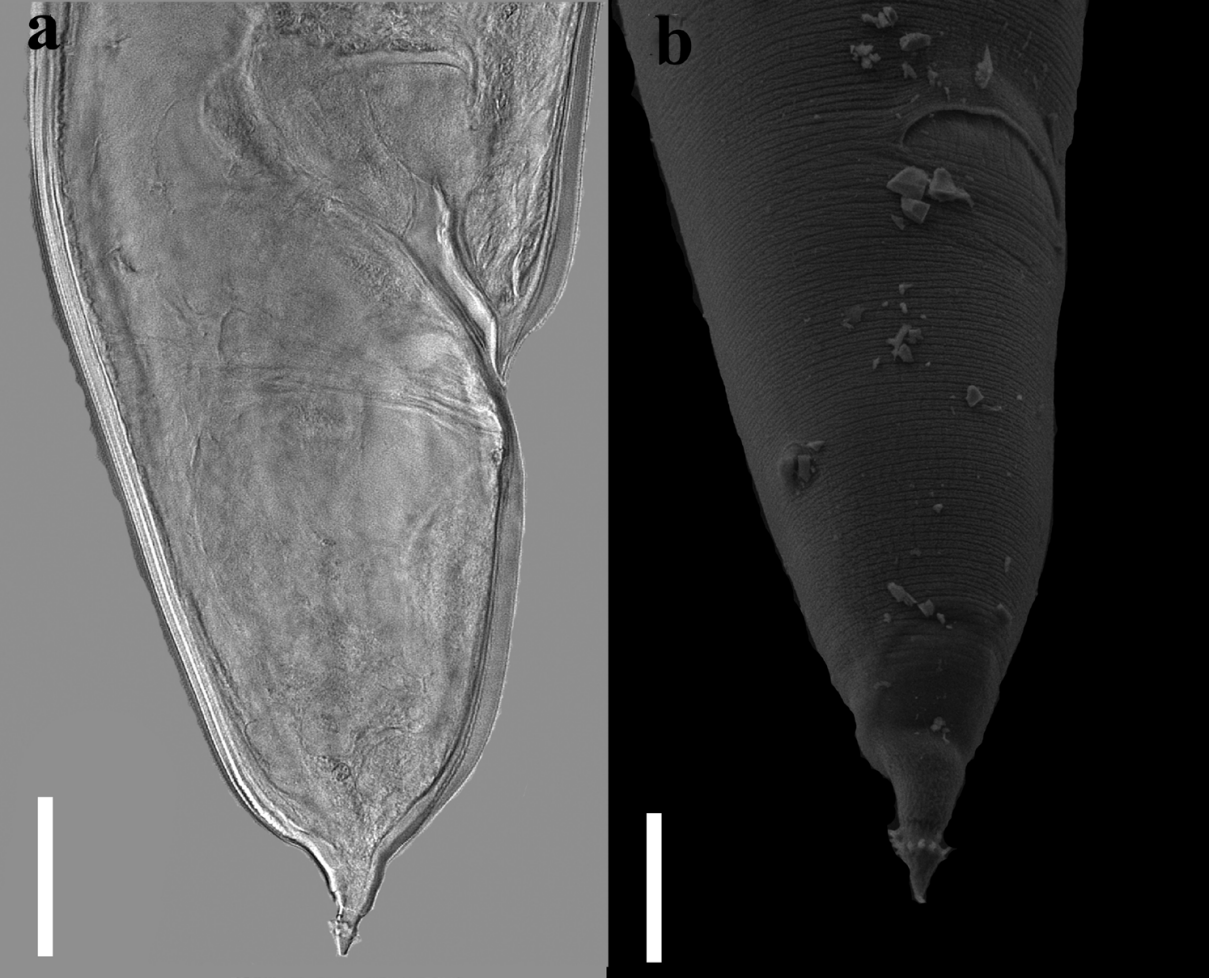
*Dichelyne* (*Dichelyne*) *jutubensis* n. sp., from *Propimelodus eigenmanni*, eastern Amazon. (a) Posterior extremity of female detail of spines; (b) Scanning electron micrographs of female detail of spines. Scale bars: (a) = 50 μm; (b) = 20 μm.

**Female (based on 15 gravid females with immature eggs, allotype measurements in parentheses):** Body length 5 mm ± 1 (4–8 mm) [4.89 mm], maximum width at the junction between esophagus and intestine 0.367 mm ± 64 (0.243–0.486 mm) [0.357 mm].. Nerve ring, deirids, and excretory pore at 0.225 mm ± 29 (0.168–0.293 mm) [0.216 mm], 0.559 mm ± 86 (0.373–0.733 mm) [0.566 mm], and 0.762 mm ± 90 (0.600–0.967 mm) [0.600 mm], respectively, from anterior end of body. Esophastome 0.269 mm ± 29 (0.202–0.313 mm) [0.276 mm] long and 0.087 mm ± 21 (0.060–0.127 mm) [0.060 mm] wide. Esophagus 0.704 mm ± 55 (0.557–0.790 mm) [0.686 mm] long and 0.117 mm ± 26 (0.067–0.153 mm) [0.123 mm] wide, representing 13% (10–17%) [14%] of total body length. Intestinal caecum of variable length 0.360 mm ± 56 (0.267–0.463 mm) [0.343 mm] long, 0.072 mm ± 17 (0.040–0.100 mm) [0.100 mm] wide, representing 51% (39–60%) [50%] of esophageal length. Vulva postequatorial, 2 mm ± 1 (1–4 mm) [2.19 mm] from anterior extremity, representing 43% (29–53%) [44%] of body length (Figures 1a, c; 2d); vulvar lips elevated. Vagina directed anteriorly from vulva. Uterus filled with numerous oval eggs with uncleaved contents 0.057 mm ± 5 (0.050–0.066 mm) long and 0.049 mm ± 3 (0.045–0.056 mm) wide. Rectum a short hyaline tube, 0.124 mm ± 26 (0.070–0.175 mm) [0.100 mm] long. Postdeirids not observed. Papilla-like phasmids laterally located in second half of tail (Figure 2e). Tail conical, 0.184 mm ± 18 (0.137–0.208 mm) [0.136 mm] long, with the presence of a crown adorned with approximately 15 small spines (Figures 1d, e; Figures 3a, b).

**Etymology:** The specific epithet *jutubensis* derives from the collecting locality of the host specimens, *i.e*., Jutuba Island in the municipality of Belém, Pará, northern Brazil.

**ZooBank Life Science Identifier (LSID):** urn:lsid:zoobank.org:pub:F1F8D162-2D79-4E5F-BFAF-1A86DBF6EF5F

**Type host:***Propimelodus eigenmanni* (Van der Stigchel, 1946) (Siluriformes: Pimelodidae).

**Site of infection:** Intestine.

**Biome:** Amazon - **Environment:** Brackish water.

**Type locality:** Marajó Bay, state of Pará, Amazon, Brazil.

**Prevalence:** 39.6% (36 infected fish/91 examined).

**Mean intensity of infection:** 2.5 nematodes per infected host (range 1–15).

## Discussion

The specimens recovered from the intestine of *Propimelodus eigenmanni* presented characteristics that are compatible with the genus *Dichelyne*, such as an elongated oral opening with a dorsoventral opening, a laterally narrowed pseudobuccal capsule (esophastome), a cephalic end equipped with four submedian papillae and a pair of lateral amphidia, as well as simple and undivided esophagus and well-developed anterior intestinal cecum (Moravec, 1998). The absence of a ventral sucker and the number of caudal papillae in males allow the present material to be allocated to the nominotypical subgenus *Dichelyne* of the cucullanid genus *Dichelyne* (Li et al., 2014).

There are currently approximately thirty species described for the subgenus *Dichelyne* (Worms, 2024), of which only five are reported for the Neotropical region: *Dichelyne* (*Dichelyne*) *spinicaudatus* Petter, 1974 (French Guiana and Argentina), *Dichelyne* (*Dichelyne*) *leporine* Petter, 1989 (Paraguay and Brazil), *Dichelyne* (*Dichelyne*) *moraveci* Petter, 1995 (Paraguay), *Dichelyne* (*Dichelyne*) *pimelodi* Moravec, Khon & Fernandes, 1997 (Brazil), and *Dichelyne* (*Dichelyne*) *bonacii* Gonzalez-Solis, Arqaez-Garcia & Guillen-Hernandez, 2002 (Mexico and Brazil), with only three recorded for Brazil.

The new species has a crown of approximately 15 small spines on its tail, which resembles *Dichelyne* (*D.*) *spinicaudatus*, a parasite of two species of perciform fish (*Centropomus undecimalis* and *Cynoscion striatus*). but this species differs from *Dichelyne* (*D.*) *jutubensis* n. sp. in the final portion of the tail, which has a conical appendage divided into 4–5 points. Additionally, the new species differs in the number of intestinal ceca (one in the new species *vs*. two in *Dichelyne* (*D.*) *spinicaudatus*) and in the distribution of caudal papillae present in males, especially the adcloacal papillae (one pair in the new species *vs*. four pairs in *Dichelyne* (*D.*) *spinicaudatus*).

Another species described for South American waters, *Dichelyne* (*D.*) *moraveci* was described parasitizing *Pseudoplastystoma fasciatum*, *Luciopimelodus pati*, and *Megalonema platano* (all Pimelodidae, Siluriformes) in Paraguay (Paraná River near Puerto El Dorado, Itapua Province), and differs in the larger size of the spicules, which reach twice the size of *Dichelyne* (*Dichelyne*) *jutubensis* n. sp. (0.547−1.093 mm long in the new species *vs*. 0.700−2.050 mm in *Dichelyne* (*D.*) *moraveci*).

*Dichelyne* (*D.*) *jutubensis* n. sp. differs from the three species found in Brazil in the morphology of the tail tip, which is adorned with spines. Morphologically, females of the new species are small (4–8 mm long), which differs from *Dichelyne* (*D.*) *leporini* (17.71–23.87 mm long), in which females are three times larger. In addition, there is a difference in the location of the deirids and excretory pore, which are located below the esophagus in the new taxon, whereas in *Dichelyne* (*D.*) *leporini* these structures are located at the same height in the middle of the esophagus. Furthermore, *Dichelyne* (*Dichelyne*) *jutubensis* n. sp. has an unpaired ventral papilla located slightly anterior to cloaca, which is absent in *Dichelyne* (*D.*) *leporini*.

The new species has ten pairs of papillae distributed in six precloacal pairs, one adcloacal pair, and three postcloacal pairs. This differs from *Dichelyne* (*D.*) *pimelodi*, which has four precloacal pairs, two adcloacal pairs, and four postcloacal pairs. In addition, *Dichelyne* (*D.*) *pimelodi* has a larger spicule and numerous ventral oblique precloacal muscle bands, a morphological characteristic absent in *Dichelyne* (*Dichelyne*) *jutubensis* n. sp.

The new species differs from *Dichelyne* (*D.*) *bonacii* in having a smaller body size in both sexes, the presence of a single intestinal cecum (two ceca are present in *Dichelyne* (*D*.) *bonacii*), and the distribution of papillae, with one pair being adcloacal and three pairs being postcloacal, while *Dichelyne* (*D.*) *bonacii* has two adcloacal pairs and two postcloacal pairs. In addition to belonging to the new taxon, it has larger spicules (0.547–1.093 mm in length in the new species versus 0.45–0.65 mm in length in *Dichelyne* (*D.*) *bonacii*). The new species is described in fish of the family Pimelodidae, in brackish waters off the northern coast of Brazil, while *Dichelyne* (*D.*) *bonacii* is recorded parasitizing two species of Lutjanidae (*Lutjanus analis* and *Rhomboplites aurorubens*) in marine waters off the northeast coast of Brazil by Alves et al. (2019). Other morphometric comparisons between the new species and other species of *Dichelyne* (*Dichelyne*) are presented in Table 1.

The Amazon Estuary comprises sections of the Amazon River subject to tidal influence, the Marajó Archipelago, and the basins flowing directly into this region from the Pará and Amapá coasts. It also encompasses an extensive coastal area strongly influenced by the Amazon River discharge (Barthem et al., 2024). Poulin (2021) describes functional biogeography as the study of the spatial patterns of biodiversity and the processes that generate them, noting that geographical patterns in parasite characteristics likely emerged in parallel with patterns in their hosts, while bioclimatic factors play a secondary role. Furthermore, Santos Reis et al. (2021) argue that fluctuating river levels, driven by rainfall intensity, and seasonal flooding of some areas likely affect host-parasite interactions and distributions. Accordingly, we propose *Dichelyne* (*Dichelyne*) *jutubensis* n. sp. as the first species of the genus identified in estuarine waters along the coast of Pará, northern Brazil. We also report *Propimelodus eigenmanni* as a new host for the genus. Our findings extend the known geographic distribution of these parasites to northern Brazil (the Brazilian Amazon).

## Data Availability

Data will be made available on request.
